# Genome and Infection Characteristics of Human Parechovirus Type 1: The Interplay between Viral Infection and Type I Interferon Antiviral System

**DOI:** 10.1371/journal.pone.0116158

**Published:** 2015-02-03

**Authors:** Jenn-Tzong Chang, Chih-Shiang Yang, Yao-Shen Chen, Bao-Chen Chen, An-Jen Chiang, Yu-Hsiang Chang, Wei-Lun Tsai, You-Sheng Lin, David Chao, Tsung-Hsien Chang

**Affiliations:** 1 Department of Biological Sciences, National Sun Yat-Sen University, Kaohsiung, Taiwan; 2 Department of Medical Education and Research, Kaohsiung Veterans General Hospital, Kaohsiung, Taiwan; 3 Department of Pediatrics; Kaohsiung Veterans General Hospital, Kaohsiung, Taiwan; 4 Department of Infectious Diseases, Kaohsiung Veterans General Hospital, Kaohsiung, Taiwan; 5 Department of Microbiology, Kaohsiung Veterans General Hospital, Kaohsiung, Taiwan; 6 Department of Obstetrics and Gynecology, Kaohsiung Veterans General Hospital, Kaohsiung, Taiwan; 7 Division of Gastroenterology, Department of Internal Medicine, Kaohsiung Veterans General Hospital, Kaohsiung, Taiwan; 8 Department of Pharmacy and Graduate Institute of Pharmaceutical Technology, Tajen University, Pingtung, Taiwan; University of Georgia, UNITED STATES

## Abstract

Human parechoviruses (HPeVs), members of the family *Picornaviridae*, are associated with severe human clinical conditions such as gastrointestinal disease, encephalitis, meningitis, respiratory disease and neonatal sepsis. A new contemporary strain of HPeV1, KVP6 (accession no. KC769584), was isolated from a clinical specimen. Full-genome alignment revealed that HPeV1 KVP6 shares high genome homology with the German strain of HPeV1, 7555312 (accession no. FM178558) and could be classified in the clade 1B group. An intertypic recombination was shown within the P2-P3 genome regions of HPeV1. Cell-type tropism test showed that T84 cells (colon carcinoma cells), A549 cells (lung carcinoma cells) and DBTRG-5MG cells (glioblastoma cells) were susceptible to HPeV1 infection, which might be relevant clinically. A facilitated cytopathic effect and increased viral titers were reached after serial viral passages in Vero cells, with viral genome mutation found in later passages. HPeV1 is sensitive to elevated temperature because 39°C incubation impaired virion production. HPeV1 induced innate immunity with phosphorylation of interferon (IFN) regulatory transcription factor 3 and production of type I IFN in A549 but not T84 cells. Furthermore, type I IFN inhibited HPeV1 production in A549 cells but not T84 cells; T84 cells may be less responsive to type I IFN stimulation. Moreover, HPeV1-infected cells showed downregulated type I IFN activation, which indicated a type I IFN evasion mechanism. The characterization of the complete genome and infection features of HPeV1 provide comprehensive information about this newly isolated HPeV1 for further diagnosis, prevention or treatment strategies.

## Introduction

Human parechovirus (HPeV), a small, round-structured, non-enveloped virus with a single-stranded and positive-sense RNA genome, belongs to the *Picornaviridae* [1]. HPeV is structurally similar to other picornaviruses because of its icosahedral symmetry and appearance on electron microscopy [2]. It was first described in 1961 as echoviruses 22 and 23 of the genus *Enterovirus* on the basis of serology and clinical presentation on identification from an outbreak of diarrhea among children [3]. However, further studies showed that properties of the virus, such as nucleotide sequence in replication and translation elements, differ from other members of the genus *Enterovirus*. So the virus was re-classified into a new genus, *Parechovirus*, and the echoviruses 22 and 23 were re-named HPeV1 and HPeV2, respectively [4,5,6]. During the past decade, several other HPeV isolates have been reported; to date, we have the full genome sequences for eight HPeV types, HPeV1 to HPeV8, and eight other types, HPeV9 to HPeV16, are known based on their viral protein 1 (VP1) sequences (http://www.picornastudygroup.com).

For replication and virus particle formation of HPeV, the open reading frame (ORF), flanked by a 5' untranslated region (5' UTR) and a 3' UTR, encodes a single polyprotein that is processed to three structural or capsid-encoding proteins (VP0, VP3 and VP1, encompassing P1) and seven nonstructural proteins (2A, 2B, 2C [P2]; 3A, 3B, 3C and 3D [P3]). Unlike other picornaviruses, HPeV VP0 nucleocapsid is not further cleaved to generate the capsid proteins VP4 and VP2 [7]. HPeV differs from members of the genus *Enterovirus* by not shutting off host cell protein synthesis during replication; its 2A protein does not likely possess protease activity to prevent normal cellular cap-dependent translation [7,8,9,10].

Many picornaviruses use immunoglobulin superfamily members or integrin of target cells as the attachment receptor to enter host cells [2]. The Arginine-Glycine-Aspartate (RGD) motif at the C-terminus of HPeV1 VP1 binds to integrin on the cell membrane as part of its entry process [11]. However, the RGD motif is absent in HPeV3, associated with neonatal sepsis and infection of the central nervous system (CNS), and in at least two types of the recently described HPeV7 and HPeV8 [12,13,14]. Mechanisms other than RGD binding to integrin may occur on infection with different HPeVs.

After virus entry, the segment located at the 5' UTR, called the internal ribosomal entry site (IRES), directs ribosomal binding to a position close to an internal start (methionine) codon [1]. There, RNA translates into a polyprotein that is subsequently cleaved by the trypsin-like protease (3C) to produce nonstructural proteins for initiation of the replication cycle of HPeV [10]. The functions of some nonstructural proteins of HPeV can only be speculated by comparison with those of picornaviruses, but 3D is known as an RNA-dependent RNA polymerase that copies viral genome during replication [7,15].

HPeV infections may be common in early childhood [16]. A large enterovirus surveillance in the United States from 1983 to 2003 revealed 73% HPeV1 infection and 68% HPeV2 infection in children < 1 year old [17]. Indeed, we have only a few reports of HPeV infection in patients > 10 years old [18,19,20]. The most common genotype of HPeV in Europe is HPeV1, followed by HPeV3 [15,21,22]. In Japan, HPeVs were isolated from 0.3% of 13,656 clinical samples collected from 1991 to 2005; the isolated HPeV types were HPeV3 (39%), HPeV1 (34.2%), HPeV6 (24.4%) and HPeV4 (2.4%) [18]. HPeV1 may be the most prevalent type worldwide. Serological ELISA revealed the seroprevalence for HPeVs from 22% to 88% in children between 2 and 24 months old, 70% in children ≤ 5 years old and 95% in adults [23]. Most infections of HPeVs are asymptomatic or clinically mild in severity in children > 5 years old and adults, and it is probably due to the age-dependent established antiviral immunity against widespread circulation and infection of HPeVs [23,24,25].

HPeV1 causes diseases in gastrointestinal and respiratory tracts, considered the primary routes of infection [25]. Nosocomial infection or outbreaks in neonate hospital departments seem to play a substantial role in both kinds of HPeV infection [26,27]. More severe consequences of HPeV infection, such as acute flaccid paralysis, myocarditis, and Reye syndrome, have been reported [13,18]; HPeV3 infection results in severe diseases of the CNS and neonatal sepsis [28]. HPeV3 infection is almost exclusively restricted to infants < 3 months old [29], which may be explained by observations of much lower seroprevalence of HPeV3 among women of childbearing age as compared with almost universal seropositivity for HPeV1 [28,30]; therefore, neonates are at increased risk of HPeV3 infection because of reduced maternal antibody protection [31]. HPeV3 differs from HPeV1 by lacking the RGD motif in VP1, so an alternative receptor may exist, thus changing the cellular tropism of HPeV3 and leading to enhanced ability to spread and replicate in the CNS [32,33].

Although HPeV is a widespread pathogen that plays a significant role in several diseases, it is not routinely detected in most laboratories because it often grows poorly in culture, typing reagents are not widely available for new types, and the technique is laborious and time-consuming [33]. Thus, we have limited information on the actual incidence of HPeV infection in clinical illnesses, and more importantly, delayed diagnosis and management in severe conditions.

In this study, we report the completed genome of a newly isolated, *in vitro*– propagated HPeV1 strain, KVP6, and its comparison with other random selected HPeV isolates with full genome information available in GenBank. We also generated a polyclonal antibody against HPeV1 VP0 to detect the cell tropism of HPeV1. The HPeV1 replication kinetics and interplay with host innate immunity were investigated. This study delineates adaptable infection systems to facilitate future investigations of the pathogenicity of HPeV.

## Materials and Methods

### Virus, cell lines and reagents

The HPeV1 KVP6 was isolated by the Virology Group, Department of Microbiology, Kaohsiung Veterans General Hospital, and propagated in Vero cells (ATCC: CCL-81) by continuous passage, at 37°C in a 5% CO_2_ atmosphere. A549 human lung adenocarcinoma cells (ATCC: CCL-185), HeLa human cervical cancer cells (Bioresource Collection and Research Center [BCRC]: 6005), and J774A.1 mouse macrophage cells (BCRC: 60140) were cultured in Dulbecco's modified Eagle's medium (DMEM) supplemented with 10% fetal bovine serum (FBS; Invitrogen). T84 colon carcinoma cells (BCRC: 60149) were grown in DMEM supplemented with 5% FBS. BHK21 hamster kidney cells (BCRC: 60041) and DBTRG-5MG human glioblastoma cells (BCRC: 60380) were cultured in RPMI 1640 medium supplemented with 5% and 10% FBS, respectively. IFNα-2a (Prospec) and IFNβ (Peprotech) were used for virus inhibition assay. PolyI:C was from Invitrogen.

### Viral titration

To determine virus titers, culture medium from HPeV1-infected cells was harvested for plaque-forming assays. Various virus dilutions were added to 6-well plates with 80% confluent Vero cells and incubated at 37°C for 2 h. After adsorption, cells were gently washed and overlaid with 1% agarose (SeaPlaque; FMC BioProducts) containing MEM supplemented with 2% FBS. After 7 days’ incubation at 37°C, cells were fixed with 10% formaldehyde, then stained with 1% crystal violet for further plaque counting. To determine the viral growth curve, 100 μl culture medium from HPeV1-infected cells in 12-well plates was harvested over time for viral titration.

### RNA extraction, viral genome sequence and quantitative real-time PCR

The RNA of the HPeV1 KVP6 isolate was extracted by the addition of 500 μl TRIzol reagent (Invitrogen), then 100 μl chloroform. The emulsion was centrifuged and the aqueous phase transferred to a fresh tube. The RNA was precipitated with 250 μl isopropanol and pelleted by centrifugation at 12,000 × g at 4°C for 10 min. Gel-like pelleted RNA was washed with 1 ml of 75% ethanol, then centrifuged at 7,500 × g at 4°C for 5 min. After removal of supernatant, it was air dried for 10 min in a laminar flow hood and resuspended in nuclease-free water at 55–60°C. cDNA was synthesized with use of a 50 mM random primer with 1 μg total RNA in a total reaction volume of 12 μl by the Superscript III reverse transcriptase method (Invitrogen).

The HPeV1 KVP6 genome sequence was accessed by 3’ RACE and then PCR with 8 pairs of oligonucleotide primers (S1 Table), which were designed on the basis of the genome sequences of HPeV1 7555312 (GenBank accession no. FM178558). All fragments of PCR products were sequenced directly in both directions and then assembled.

qPCR amplification involved 6 ng cDNA in 10 μl SYBR green PCR master mix (Applied Biosystems) with 3 μM primers in the ABI StepOne Plus Real-Time PCR System (Applied Biosystems). The relative gene mRNA expression was normalized to that of glyceraldehyde 3-phosphate dehydrogenase (GADPH) as a loading control. The primer sequences for qPCR are in supplemental material S2 Table.

### Phylogenetic analysis and similarity plots

The random selected HPeV genome sequences for phylogenetic and similarity plots analysis were HPeV type 1 strains Harris (accession no. L02971), SH1 (accession no. FJ840477), and 7555312 (accession no. FM178558); HPeV type 2 strain Williamson (accession no. AJ005695); HPeV type 3 strains Can82853-01 (accession no. AJ889918) and A308/99 (accession no. AB084913); HPeV type 4 strains K251176-02 (accession no. DQ315670) and Fuk2005-123 (accession no. AB433629); HPeV type 5 strains T92-15 (accession no. AM235749) and CT86-6760 (accession no. AF055846) [34]; HPeV type 6 strains NII561-2000 (accession no. AB252582) and 2005–823 (accession no. EU077518); HPeV type 7 strain PAK5045 (accession no. EU556224); HPeV type 8 strain BR/217/2006 (accession no. EU716175); and Ljungan virus (accession no. EF202833). The nucleotide and translated amino acid sequences of HPeV1 KVP6 and other reference HPeV strains were aligned by use of MEGA 5.2.2. The resulting phylogenetic trees were constructed by the neighbor-joining method with 1000 replication for bootstrapping and pairwise deletion as gap-missing data-processing. Similarity analysis involved the known full-length nucleotide sequences of HPeV1 to HPeV8 genomes against HPeV1 KVP6, and plots were generated by use of SimPlot 3.5.

### Antibodies

The Pep VP0-21, a 21-amino-acid synthetic peptide, NLTQHPSAPTIPFTPDFRNVD, derived from the conserved VP0 caspid protein, was found highly antigenic for detection of HPeV [23]. New Zealand rabbits were vaccinated with Pep VP0-21 peptide in six boosts over 2 months. At 7 days after the last boost, immunized rabbit serum containing anti-HPeV1 VP0 antibody was harvested and peptide affinity-purified. The antibodies for phospho-IRF3 (pS386) (#2562-1, Epitomics), IRF3 (#sc-9082, Santa Cruz Biotechnology), phospho-STAT1 (phospho-Tyr701) (GTX50118, GeneTex), STAT1 (#9172, Cell Signaling) and β-actin (MAB1501, Millipore) were used for immunoblotting.

### Immunofluorescence assay

Mock- or virus-infected cells were fixed in 4% paraformaldehyde for 30 min, washed with phosphate-buffered saline (PBS) 3 times, then permeabilized with 0.5% Triton X-100 for 10 min. After three washes with PBS, cells were blocked with 10% skim milk in PBS for 60 min. Cells were incubated with an anti-HPeV VP0 antibody (1:500) at 4°C overnight, then secondary antibody Alexa Fluor-568 goat anti-rabbit IgG (1:1,000, Invitrogen) at room temperature for 2 h. Cell nuclei were stained with 4',6-diami-dino-2-phenylindole (DAPI) (1 mg/ml, 1:100,000 dilution in PBS) for 10 min at room temperature. After three washes with PBS, cells were examined under a fluorescence microscope.

### Immunoblotting analysis

Cells were lysed in RIPA buffer (150 mM NaCl, 0.5% sodium deoxycholate, 1% NP40, 0.1% SDS, 50 mM Tris-HCl [pH 8.0]) containing protease inhibitor (Roche). Harvested cell extracts were separated by 10% SDS-PAGE and transferred to PVDF membranes, which were incubated with primary antibody, then horseradish peroxidase-conjugated secondary antibody (Jackson ImmunoResearch Laboratory) and visualized by an enhanced chemiluminescence system (Thermo). Images were acquired by use of BioSpectrum Image System (UVP, Upland, CA).

### Cell proliferation assay

WST-1 assay (Roche) was used to monitor cell proliferation; A549 and T84 cells were trypsinized and resuspended in culture medium, then plated at 5×10^3^ cells per well in 96-well plates and incubated overnight, then incubated with 10 μl WST-1 reagent (Roche) for 2 h. The cell viability was quantified by multi-well spectrophotometry (Anthos). The absorbance at 450 nm was monitored and the reference wavelength was set at 620 nm.

### Luciferase reporter assay

TurboFect transfection reagent (Thermo Scientific) was used for transient transfection following the manufacturer’s protocol. Cells cultured in 12-well plates were transfected with IFN-stimulated response element (ISRE) Luc reporter plasmid before viral infection or IFNβ stimulation. pRL-TK (Promega), encoding *Renilla* luciferase under an HSV thymidine kinase promoter, was an internal control. Cell lysates were collected for dual-luciferase assay (Promega). Firefly luciferase activity was normalized to that of *Renilla* luciferase.

## Results

### HPeV1 KVP6 genome sequence and analysis

The complete genome of the HPeV1 virus contains 7,329 nucleotides, excluding the 3' poly(A) tail. Flanked by a 681-nt 5' UTR and a 111-nt 3' UTR, the predicted polyprotein is encoded by a 6,537-nt single ORF. The genome organization of HPeV1 KVP6 is identical to that of other HPeVs [7,15] (S3 Table). The genome sequence of HPeV1 KVP6 has been deposited in GenBank (accession no. KC769584).

**Figure 1:**
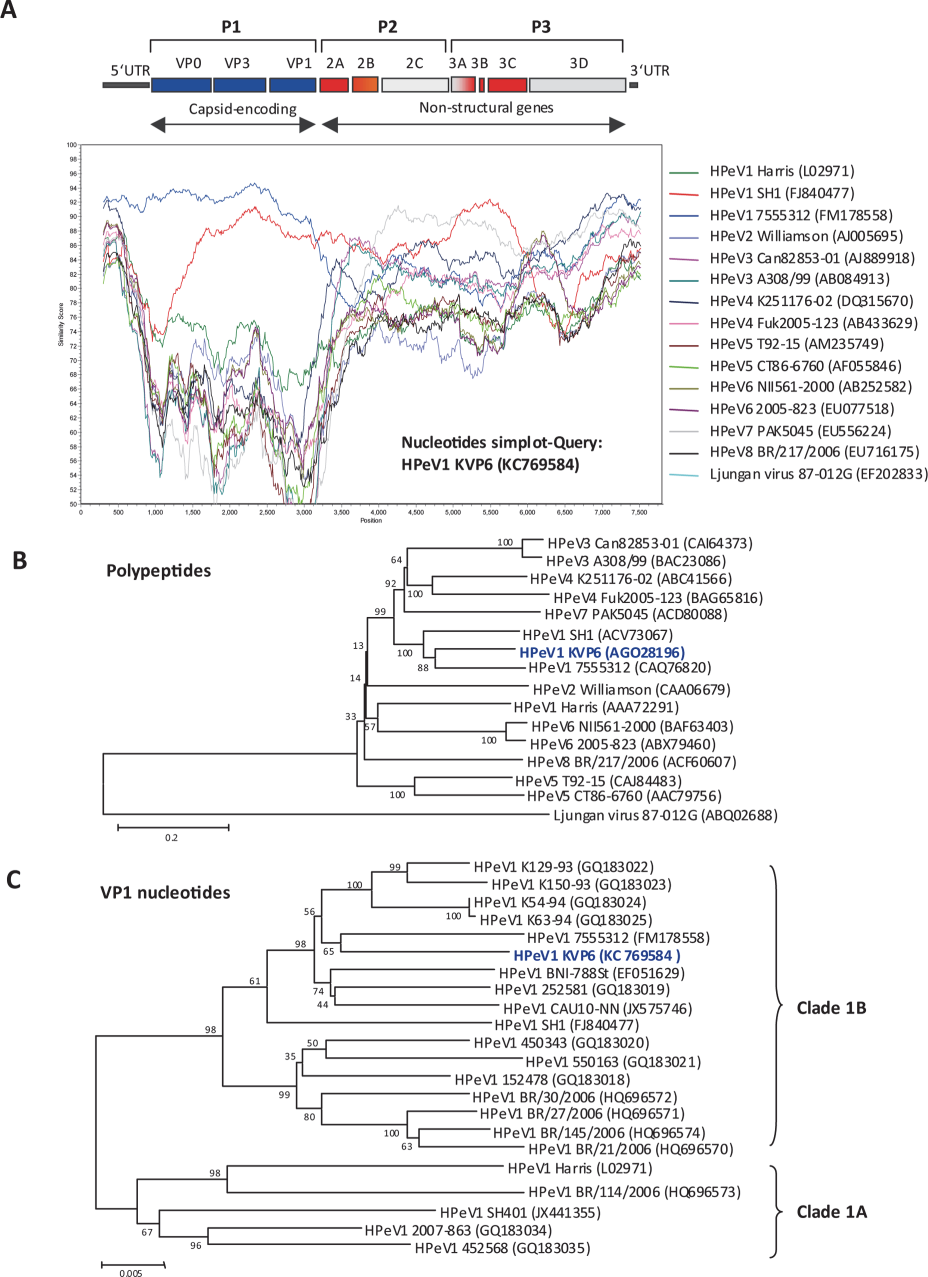
HPeV1 KVP6 full-genome analysis. **(A)** SimPlot analysis of complete genome sequence of HPeV1 KVP6 with other strains or types of HPeV genomes. **(B)** Phylogenetic analysis of HPeV1 KVP6 full-length polypeptide with other HPeVs. **(C)** Phylogenetic analysis of VP1 nucleotide fragment of HPeV KVP6 with other strains of HPeV1. The clade of HPeV1 is indicated. The genome or protein accession number in GenBank is indicated.

We aligned the entire genome nucleotide sequence of HPeV1 KVP6 with the 14 completed genomes of HPeV1-8 and that of Ljungan virus, a rodent parechovirus. We performed a SimPlot analysis on these known HPeV full-length nucleotide sequences against HPeV1 KVP6 to identify a possible recombination event between different HPeV prototypes. The SimPlot analysis revealed the highest similarity of HPeV1 KVP6 with HPeV1 strain 7555312 in the highly variable capsid-encoding regions VP0, VP3 and VP1, so HPeV1 KVP6 grouped with HPeV1 [35,36] (Fig. 1A); however, in the more conserved regions, such as 2C and 3D, HPeV1 KVP6 had higher similarity with HPeV7 PAK5045. Such findings may have resulted from a recombination event during HPeV evolution [35]. In addition, full genome alignment showed that HPeV1 KVP6 shared the highest nucleotide sequence homology, 87%, with the HPeV1 strain 7555312 and a mean of 77% identity with other HPeV isolates (S4 Table). At the polyprotein level, all isolates possessed a mean of 88% amino acid identity; however, HPeV1 KVP6 shared 97% amino acid homology with HPeV1 strain 7555312 (Fig. 1B and S5 Table). Phylogenetic analysis of structural regions, in particular of VP1 protein because of its decisive role in molecular typing of HPeV [34], showed the HPeV1 KVP6 VP1 sequence close to that of HPeV1 strain 7555312 (Fig. 1C), and HPeV1 KVP6 can be typed to HPeV1 clade 1B [35]. In fact, similar findings were noted in the phylogenetic analysis of nonstructural regions (data not shown).

### Cell type tropism of HPeV1

**Figure 2:**
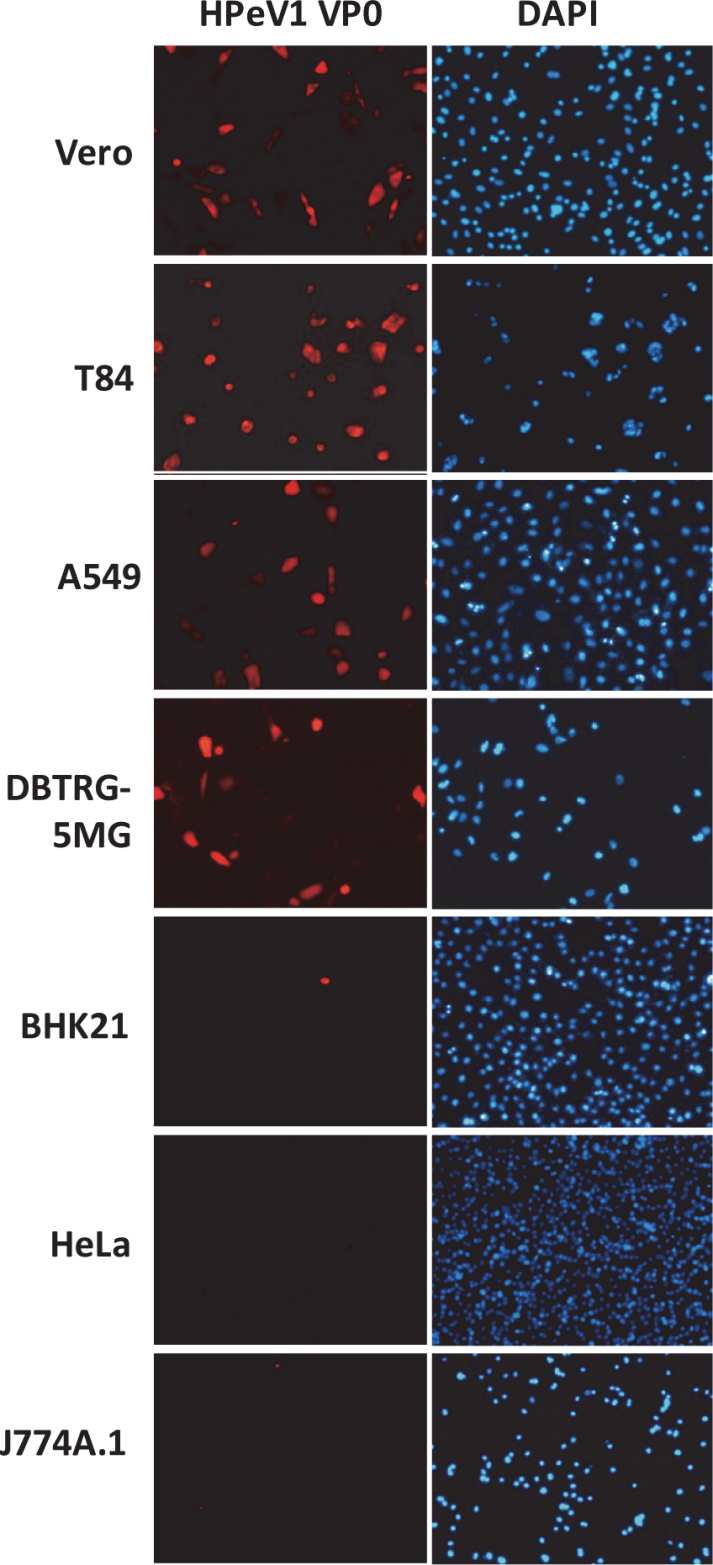
Cell tropism of HPeV1. Vero, T84, A549, DBTRG-5MG, BHK21, HeLa and J774A.1 cells at 2×10^5^ were infected with HPeV1 for 6 h at multiplicity of infection (MOI) = 1; HPeV1 VP0 was detected by immunofluorescence assay with anti-VP0 antibody; images show the red fluorescence of VP0 staining in susceptible cell types. DAPI staining indicated cell nucleus.

To characterize the infection features of this newly isolated HPeV1, we tested the cell type tropism of HPeV1; various cell types were infected with HPeV1, and their susceptibility to infection was measured by immunofluorescence assay with anti-HPeV1 VP0 antibody. Vero cells (monkey kidney epithelial cells) are widely used in virology and show susceptibility for HPeV1 infection, which could be a positive control for infection. In addition, T84 cells (intestinal carcinoma), A549 cells (lung carcinoma cells) and DBTRG-5MG (glioblastoma cells) were susceptible to HPeV infection; BHK21 cells (baby hamster kindney epithelial cells), HeLa cells (human cervival cancer cells) and J774A.1 cells (mouse macrophages) were not infected with HPeV1 (Fig. 2). Because HPeV1 infection causes clinical symptoms including respiratory- and gastrointestinal-tract and CNS symptoms [25], the cell tropism results might reflect in part an association of HPeV1 infection and its target organs. Our data also support the cell tropism of HPeV1 Harris strain and other clinically isolated HPeV1 strains [31,37].

### HPeV1 progeny analysis and the kinetics of replication

**Figure 3:**
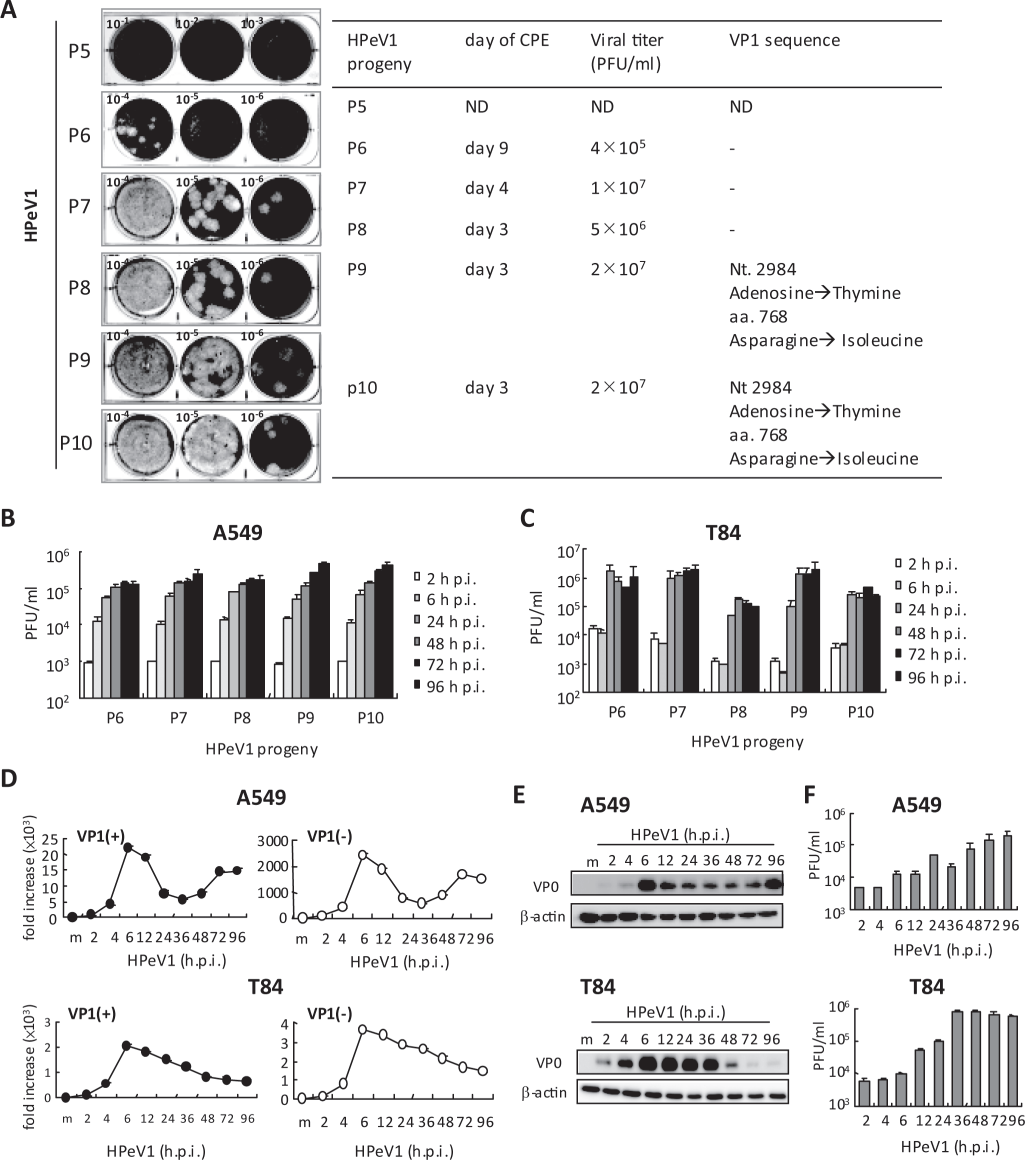
HPeV1 propagation and replication analysis. **(A)** Left panel, plaque-forming assay of HPeV1 titration of P5-P10 progeny. Right panel, cytopathic effect (CPE) formation, HPeV1 viral titer, and the results of VP1 sequence for each progeny. P5, ND: not determined; P6-P8, sequence no change; P9-P10, the point nucleotide and amino acid mutation are indicated. **(B)** A549 cells and **(C)** T84 cells were infected with each progeny at MOI = 1; plaque-forming assay of virus production kinetics in culture medium harvested at indicated times. **(D)** RT-qPCR analysis of mRNA level of HPeV1 structure-protein (VP1) positive sense (+) and negative sense (-) in A549 cells (2×10^5^, upper panels) and T84 cells (2×10^5^, lower panels) infected with HPeV1 (MOI = 1). Data are mean±SD. **(E)** Immunoblotting of VP0 viral protein and loading control, β-actin, in A549 cells (2×10^5^, upper panels) and T84 cells (2×10^5^, lower panels) infected with HPeV1 (MOI = 1) as indicated. **(F)** A549 cells (2×10^5^, upper panel) and T84 cells (2×10^5^, lower panel) were infected with HPeV1 (MOI = 1); plaque-forming assay of virus production in culture supernatant at the indicated times. Data are mean±SD.

To understand whether viral features of the cytopathic effect (CPE) and viral genome are changed during viral amplification, we propagated the HPeV1 KVP6 virus from the 4^th^ progeny (P4) in Vero cells with serial sub-passaging. We found no CPE at post-infection day 10 in P5 infected cells, which might due to the extremely low virion production. Nevertheless, P6 progeny showed CPE and plaque formation; CPE was accelerated from day 9 to 3 in the later progenies, and viral titer was increased at P9 and P10 (Fig. 3A, left panels). Genome mutation during virus passage was reported in hepatitis A and E virus infection and severe acute respiratory syndrome (SARS-CoV) infection [38,39,40]. We analyzed the VP1 sequence from different progenies, because in P9 of HPeV1 KVP6, the nucleotide position 2984 adenosine was replaced by thymine and resulted in a mutation in amino acid position 768 from Asparagine to Isoleucine (Fig. 3A, right panel). Because the amino acid position 768 is close to the RGD motif (position 763~765), whether this mutation relates to receptor binding might be of interest. Mutations other than the 2984 positive one may exist in other regions of the genome. These data suggest that HPeV1 KVP6 virulence and viral titer could be amplified by serial passage *in vitro*; however, the genome instability would appear during the passages.

We determined the infection and viral replication activities of P6 to P10 progeny of HPeV1 KVP6 in A549 and T84 cells until CPE appeared at 96 h post-infection (hpi). In both cell types, these progenies showed similar kinetics of production of assembled viral particles, with viral titer amplified approximately 100-fold at 24 hpi, then gradually increased in A549 cells during incubation (Fig. 3B). In T84 cells, after 24 hpi, the virus was sustained at a constant level until 96 hpi (Fig. 3C). The virus replication pattern also suggested that HPeV1 might go through one complete cycle during 2 and 6 hpi in A549 cells and 6 and 24 hpi in T84 cells. According to the applicable viral titer and genome stability, HPeV1 KVP6 P7 progeny were used for further study.

In A549 cells, for HPeV1 viral RNA replication kinetics, RT-qPCR analyses of HPeV1 viral genes showed two peaks of positive- and negative-sense RNA gene expression of VP1. The first peak occurred at 6 hpi and the second but smaller surge at 72 or 96 hpi (Fig. 3D, upper panels). The two-peaks phenomenon of HPeV1 replication in A549 cells was further supported by VP0 immunoblotting analysis (Fig. 3E, upper panels). The data showed the two rounds of viral replication in A549 cells, the first round during 2 to 6 hpi and the second during 72 to 96 hpi. However, the two peaks of VP1 replication in A549 cells was not found in T84 cells: the mRNA and protein expression of HPeV1 VP1 peaked at 6 hpi, then decreased over time (Fig. 3D and E, lower panels). As well, the fold increase in VP1 RNA was lower in T84 than A549 cells.

The kinetics of viral production was assayed by plaque-forming assay. In A549 cells, the HPeV1 accumulated over time and reached > 10^5^ PFU/ml at 72 or 96 hpi, approximately 10-fold higher than at 6 hpi, (Fig. 3F, upper panel), with approximately 100-fold increase in T84 cells (Fig. 3F, lower panel). Thus, T84 cells might be more susceptible to HPeV1 producing a higher level of virions than A549 cells. These viral titer findings also suggested that the virion production kinetics was not associated with the dynamic mRNA and protein expression in infected cells. However, why the second round of viral gene or protein expression did not proceed in T84 cells during 72 to 96 hpi and why the relatively low mRNA level in T84 cells is unclear. Whether these phenomena are cell type-dependent requires further investigation.

### The effect of incubation temperature on HPeV1 replication

**Figure 4:**
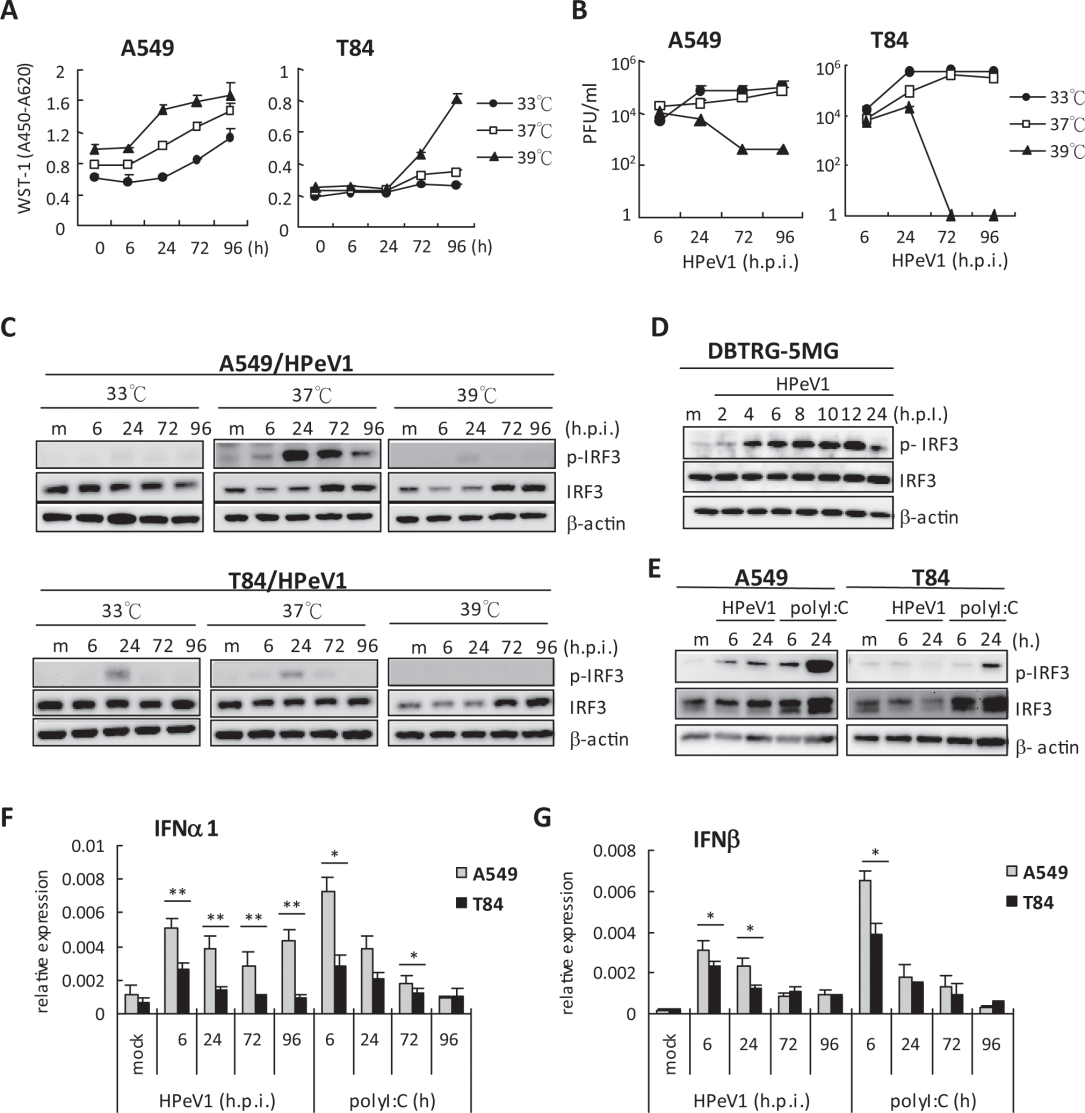
The effect of temperature on HPeV1 replication and innate immunity. **(A)** WST-1 cell proliferation assay in A549 and T84 cells (1x10^4^) at 33, 37 and 39℃ culture conditions. Data are mean±SD. **(B)** HPeV1 virus titration measured from HPeV1 (MOI = 1) infected A549 and T84 cells (2×10^5^) at different culture temperatures. Data are mean±SD. **(C)** Immunoblotting of phospho-IRF3, total IRF3 and -actin in cell lysates harvested from HPeV1 infected A549 cells (upper panels) and T84 cells (lower panels) or DBTRG-5MG cells **(D)** at indicated times and temperatures. **(E)** Immunoblotting analysis of phospho-IRF3, total IRF3 and β-actin expression in A549 or T84 cells (2×10^5^) infected by HPeV1 (MOI = 1) or transfected with polyI:C (2 μg). **(F and G)** RT-qPCR analysis of IFNα and IFNμ mRNA expression in A549 or T84 cells infected by HPeV1 (MOI = 1) or stimulated by poly I:C (2 μg) at indicated times. Data are mean ± SD from 3 independent tests. Student *t*-test, * *p*<0.05, ** *p*<0.01.

WST-1 assay demonstrated that increasing culture temperature promoted A549 and T84 cell proliferation (Fig. 4A). Although the abnormal physical temperature 33°C and 39°C did not impair cell viability within 4 days (Fig. 4A), HPeV1 virion production was inhibited at 39°C incubation at 24 hpi in A549 and T84 cells (Fig. 4B). To test the HPeV1 virion stability in different temperatures, HPeV1 virus was incubated at 33°C, 37°C and 39°C between 6 and 48 h before infection of A549 and T84 cells. Immunofluorescence assay revealed that HPeV1 infectivity was impaired after 24-h incubation under all incubation conditions. HPeV1 infection with 6-h short-term incubation at 33°C and 37°C retained the infectivity of the positive control; nevertheless, 39°C-treated virus could not efficiently infect A549 and T84 cells (S1A and B Figs.), which suggested the HPeV1 is unstable at 39°C. HPeV1 virion may be sensitive to elevated temperature.

Host innate immune response is critical against virus; therefore, we examined whether temperature affected viral infection-mediated innate immune activation. Interferon (IFN) regulatory factor 3 (IRF3) plays a key role in virus triggering innate immunity activation; it coordinates with NFκB p65 subunit and c-Jun/AP1 to bind the type I IFN promoter region to induce type I IFN transcription [41,42,43]. Thus, we monitored IRF3 phosphorylation to understand the HPeV1-mediated innate immune response under different incubation temperatures. HPeV1 induced high-level and long-term IRF3 phosphorylation in A549 cells at 37°C but not 39°C culture (Fig. 4C, upper panels); virus production failed at 39°C (Fig. 4B). The findings of 37°C and 39°C incubation might reflect a typical viral load–dependent response of IRF3 activation under normal physical temperature. Additionally, 33°C is lower than 37°C, and A549 cells showed defective IRF3 activation, even though HPeV1 was produced at 33°C incubation (Fig. 4B), which suggested the low host response to virus infection at 33°C. In fact, polyI:C-induced IRF3 phosphorylation level was lower in 33°C than 37°C in A549 and T84 cells (S2 Fig.). Virus-mediated IRF3 activation was ambiguous at all three culture temperatures in T84 cells (Fig. 4C, lower panels); more HPeV1 virions were produced in T84 than A549 cells at 33°C and 37°C (Fig. 4B). We also detected IRF3 activation in another HPeV1-susceptible cell line, DBTRG-5MG glioblastoma cells, which showed a similar IRF3 activation pattern as for HPeV1-infected A549 cells (Fig. 4D).

The diversity of HPeV1-mediated IRF3 activation in different cell types was supported by double-stranded RNA polyI:C stimulation inducing greater phosphorylation of IRF3 in A549 than T84 cells (Fig. 4E); consistently, HPeV1 and polyI:C induced a higher mRNA level of IFNα1 and IFNβ in A549 cells (Fig. 4F and G). As compared with A549 cells, T84 cells showed a more torpid innate immune response. Cell type might be a critical factor in viruses triggering antiviral signaling activation.

### Cell type-dependence of efficiency of type I IFN against HPeV1

**Figure 5:**
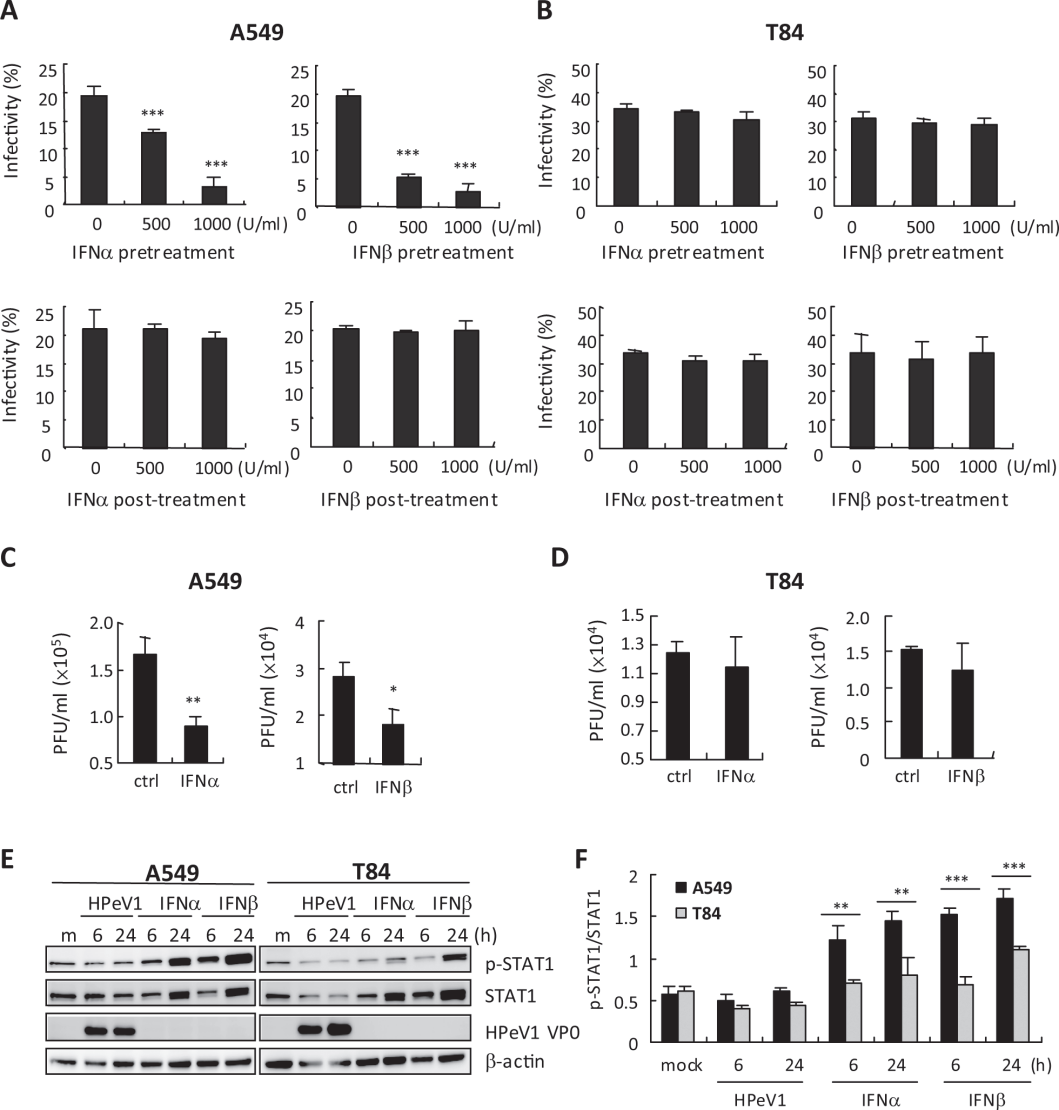
Cell type-dependence of type I IFN anti HPeV1 activity. **(A)** Upper panels, immunofluorescence assay with anti-VP0 antibody of 2×10^5^ A549 cells and **(B)** T84 cells treated with IFNα;-2a or IFNβ (500 and 1000 U/ml) for 24 h before HPeV1 (MOI = 1) infection for 6 h. Lower panels, type I IFN was added after HPeV1 adsorption; then infected cells were analyzed at 6 hpi. Red fluorescence of VP0 staining cells was quantified and calculated with DAPI nuclear staining signaling to show infectivity. Data are mean ± SD from 3 observed fields. ***p<0.*001* compared with control. **(C and D)** A549 and T84 cells (2×10^5^) were treated with IFNα-2a or IFNβ (1000 U/ml) for 24 h before HPeV1 infection (MOI = 1). Plaque forming assay of HPeV1 viral titer at 6 hpi. Data are mean ± SD. Student *t* test, * *p*<0.05, ** *p*<0.01 compared with control. **(E)** Immunoblotting of phospho-STAT1, total STAT1 and β-actin in A549 and T84 cells (2×10^5^) with IFNα-2a or IFN (1000 U/ml) treatment for 24 h. **(F)** Immunoblots of arbitrary unit of pSTAT1/STAT1 of A549 cells and T84 cells.

To understand the efficiency of type I IFN against HPeV1, A549 and T84 cells were treated with IFNα-2a or IFNβ before or after HPeV1 infection, and infectivity was analyzed by immunofluorescence assay (S3 Fig.). The infection rate of HPeV1 was estimated. IFNα-2a and IFNβ pretreatment reduced HPeV1 infection, but this effect was not shown with IFN post-treatment in A549 cells (Fig. 5A). Surprisingly, T84 cells showed no anti-HPeV1 activity of type I IFN with pretreatment or post-treatment with IFN (Fig. 5B). Viral titration assay also revealed the effect of type I IFN against HPeV1 in A549 but not T84 cells (Fig. 5C and D). Therefore, we measured the level of Janus kinase (JAK)-signal transducer and activator of transcription 1 (STAT1) activation of the type I IFN signaling pathway. As compared with T84 cells, A549 cells showed that IFNα-2a and IFNβ induced higher levels of STAT1 and phosphorylated STAT1 (Fig. 5E and F). These data would explain in part that pretreatment with type I IFN inhibited HPeV1 infection in A549 but not T84 cells and suggested that the efficiency of type I IFN against HPeV1 was cell type–dependent. Interestingly, HPeV1 was not able to induce STAT1 activation in either cell type (Fig. 5E and F), which may be important for HPeV1 infection via modulating the IFN signaling pathway.

### HPeV1 attenuates type I IFN signaling

**Figure 6:**
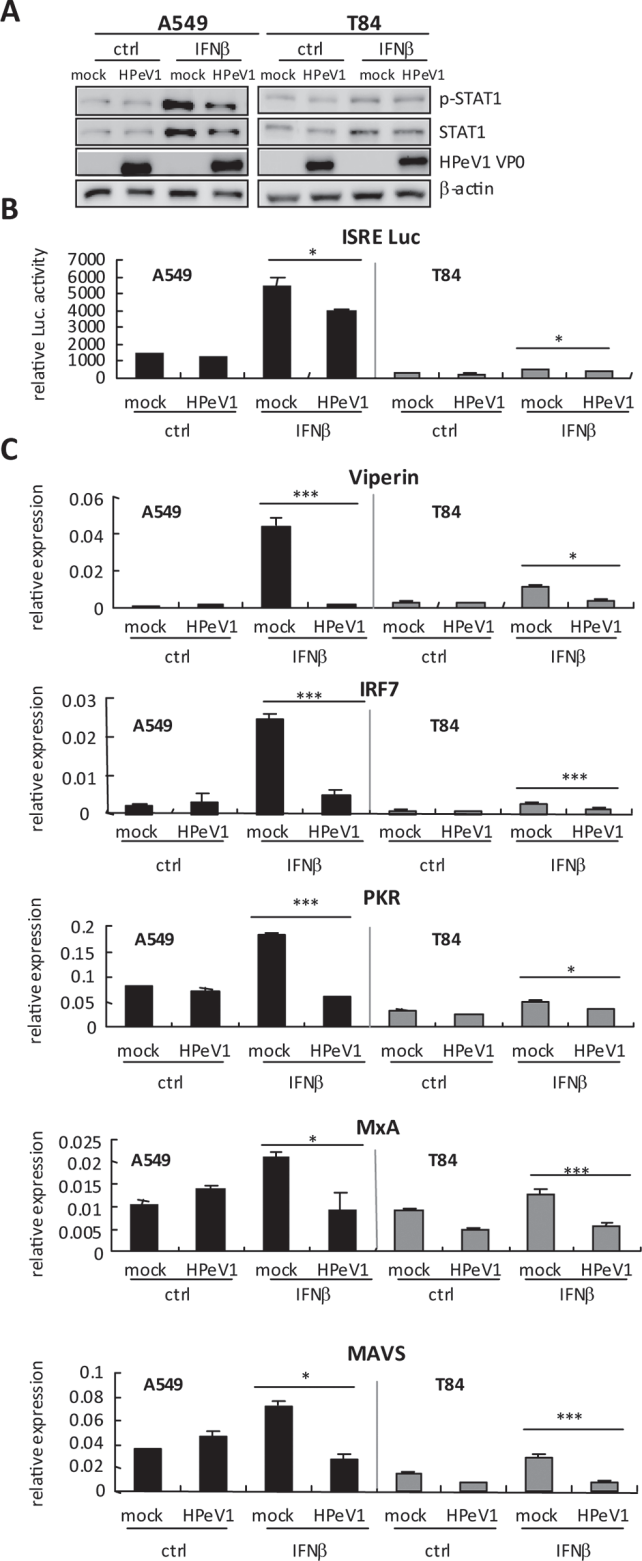
HPeV1 downregulates type I IFN activity. **(A)** A549 and T84 cells (3×10^5^) were infected with mock or HPeV1 at MOI = 5 for 6 h, then treated with IFNβ (1000 U/ml) for 18 h; untreated cells are indicated as ctrl. Immunoblotting of levels of phospho-STAT1 and total STAT1 in whole cell extracts. HPeV1 VP0 indicates viral infection and β-actin is a loading control. **(B)** Interferon-stimulated response element (ISRE) reporter assay was performed in A549 and T84 cells (3×10^5^) transfected with ISRE-luciferase reporter plasmids (400 ng) and control vector pRL-TK (40 ng) for 24 h, then infected with HPeV1 for 6 h. After 24 h of IFNβ (1000 U/ml) stimulation, ISRE luciferase activity was measured by dual-luciferase assay and **(C)** RT-qPCR of mRNA expression. Data are mean ± SD of at least 3 independent experiments. Student *t* test, * *p*<0.05, *** *p*<0.005.

Our results indicated that type I IFN post-treatment could not inhibit HPeV1 infection and HPeV1 failed to activate STAT1 (Fig. 5); HPeV1 might feature a type I IFN evasion machinery, which was described in other RNA virus infection models [44,45,46]. Type I IFN-activated STAT1 phosphorylation was reduced in HPeV1-infected A549 cells (Fig. 6A, left panel), with a slight difference in T84 cells (Fig. 6B, right panel), which might due to the low response to type I IFN of T84 cells. Another more sensitive assay revealed that HPeV1 attenuated the reporter luciferase activity of type I ISRE (Fig. 6B). Consistently, IFNβ induced the mRNA expression of antiviral-associated proteins such as Viperin, IRF7, PKR, MxA and MAVS, which was significantly inhibited by HPeV1 in A549 and T84 cells (Fig. 6C). These results suggested that HPeV1 infection interfered in type I IFN signaling activation. The evasion of an antiviral mechanism would be important for HPeV1 replication, which also supported the inefficient anti-HPeV1 activity with type I IFN post-treatment (Fig. 5A and B, lower panels).

## Discussion

HPeVs cause various symptoms ranging from mild diarrhea to sepsis and meningitis among young children; however, because HPeVs are difficult to propagate in cultured cells, the pathological mechanism of HPeVs remains largely unknown. We established a propagation and infection model of a clinically isolated domestic HPeV1 virus, a newly isolated Taiwanese strain HPeV1 KVP6, which allowed us to characterize the genome and infection features of the virus. We found genome intertypic recombination between HPeV1 KVP6 and HPeV7 in the nonstructural coding region, which might be associated with HPeV evolution and pathogenesis. *In vitro* study showed impaired HPeV1 replication at 39°C culture, so physically high temperature restricts HPeV1 replication. The HPeV1 infection induced an inconsistent level of innate immune responses in different cell types. We found viral-induced IRF3 activation and increased level of type I IFN production in A549 lung carcinoma cells but not T84 colon carcinoma cells; in particular, type I IFN treatment failed to inhibit HPeV1 infection in T84 cells. Our findings may explain why patients with HPeV1 infection frequently show gastrointestinitis symptoms. Furthermore, in both A549 and T84 cells, HPeV1 downregulated the activation of STAT1, downstream of IFN, and antiviral gene expression; this innate immune evasion should be critical for HPeV1 infection.

### HPeV1 KVP6 genome recombination

SimPlot analysis showed that KVP6 had high similarity with HPeV1 strain 7555312 and SH1 but not HPeV1 prototype Harris strain. However, after the VP1 region, the similarity between KVP6 and 7555312 strains decreased, with more similarity with HPeV7 PAK5045 in 2C and HPeV4 K251176-02 in 3D of the nonstructural regions, which suggested intertypic recombination events [35,36]. The finding would support that the most frequent breaking points for recombination flanked the capsid-encoding region [35,36,47]. In addition, HPeV5 and HPeV6 showed recombination at the viral genome P2-P3 junction, within the 2C [48,49]. The C-terminus of VP1 of most HPeVs contained the RGD receptor-binding domain, which is critical for cell entry [11], and recombination at this position could thus have important evolutionary mechanisms in HPeVs [35,36]. Previous reports indicated frequent recombination among HPeV1, -4, -5 and -6 but more restricted among HPeV3 strains [35]; of interest, HPeV3 lacks an RGD motif in the VP1 C-terminus [12].

### HPeV1 replication kinetics is affected by incubation temperature

Viral replication kinetics indicated that viral genes were highly expressed in “two-peaks” at 6 and 72 hpi in A549 cells, and “one-peak” at 6 hpi in T84 cells; however this phenomenon might be not related to HPeV1 production, which gradually accumulated in a time-dependent manner; whether the difference depends on cell type needs to be investigated further. In addition, high incubation temperature such as 39°C, considered a febrile status in humans, might inhibit HPeV1 virion production. Similar findings were reported in enterovirus 70 and coxsackievirus A24, also members of the *Picornaviridae* family, in that replication was reduced with increased temperature [50]. In this study, we observed viral gene-expression shutdown with high temperature, which suggested that viral RNA replication was attenuated at high temperature. A possible mechanism of temperature-regulated viral gene replication was described in the infection of influenza A virus, a single-stranded and negative-sense genomic RNA virus. The viral polymerase is dissociated from the viral genome promoter at high temperature (41°C), which leads to failed genomic replication [51]. Moreover, we found that HPeV1 infectivity was retained at 33°C and 37°C but impaired at 39°C incubation before cell inoculation, which suggests the instability of HPeV1 viral particle in elevated temperature.

### The innate immune response of HPeV1-infected cells

Following virus infection, antiviral innate immune responses are initiated in host cells. The transcription of type I IFN, mainly IFNβ, is rapidly induced via cooperative binding to its promoter by three families of transcription factors: NF-κB, activator protein 1 (AP1) and IRF [52,53]. The secreted IFNβ acts on neighboring cells and activates the STAT pathway via type I IFN receptor to induce IRF7 expression, which may result in amplification of type I IFN induction via such a positive feedback mechanism [54]. Among the members of IRFs, IRF3 is critically involved in the initial induction of IFNβ when cells are infected by viruses [54]. Although we found that in A549 cells, HPeV1 induced a sustained level of total IRF3 at both 37°C and 39°C, phosphorylated IRF3, the activated form of IRF3, only emerged and lasted until late infection at 37°C, not 39°C. From the results we just mentioned, high incubation temperature inhibited HPeV1 production, especially during late infection. However, at 33°C, productive HPeV1 did not induce IRF3 phosphorylation in both A549 and T84 cells. Therefore, the activation of IRF3 may be strongly correlated with viral load and also temperature. Our other infection model showed the phenomenon of HPeV-dependent IRF3 activation [41]. Moreover, the effect of type I IFN against HPeV1 was inconsistent between A549 and T84 cells in that type I IFN pretreatment inhibited HPeV1 infection in A549 but not T84 cells perhaps because of the lower sensitivity of type I IFN signaling in T84 cells.

To establish a pathogenic infection, modulation of type I IFN activity occurs in many viruses [41,44,45,55]. In particular, picornaviruses, such as poliovirus, enterovirus, foot-and-mouth disease virus, hepatitis A virus, rhinovirus, and coxsackievirus B3, can block RIG-I-, MDA5- and MAVS-mediated type I IFN production by viral protease activity [56,57,58,59,60,61]. Investigating the mechanism of HPeV1-blocked IRF3-mediated type I IFN production in T84 cells would be interesting. Previous study revealed enterovirus 71 2A protease reduces IFN receptor 1 activity to disrupt the activation of STAT1, STAT2, Jak1 and Tyk2 [46]. These data may suggest future investigation of HPeV1 regulating type I IFN activity because we also found the HPeV1 can attenuate type I IFN downstream signaling and antiviral protein expression.

HPeV1 KPV6 is the first isolation from Taiwan. This study supplies information for understanding the genome characteristics and the fundamental infection features of this newly isolated virus. Our findings may be beneficial for the HPeV1 evolution analysis, viral detection or treatment in laboratory or clinical practice. Furthermore, the domestic surveillance and clinical significance of HPeV infection in Taiwan remains to be explored. With the advent of diagnostic tools, evaluating the impact of HPeV infection in pediatric or even newborn populations is important.

## Supporting Information

S1 Fig The effect of incubation temperature on HPeV1 stability. HPeV1 viral stocks were incubated at 33°C, 37°C and 39°C for 6, 24, 36 and 48 h before inoculation of A549 cells **(A)** and T84 cells **(B)**. Immunofluorescence assay with anti-VP0 antibody at 6 h post-infection (hpi) of HPeV1 infection (multiplicity of infection [MOI] = 5). The positive controls (upper panels of A, B) are cells infected with HPeV1 without pre-incubation.
(TIF)

S2 Fig PolyI:C stimulates IRF3 activation at 33°C and 37°C incubation. Immunoblotting analysis of phospho-and total IRF3 in 2×10^5^ A549 **(A)** or T84 cells **(B)** transfected with polyI:C (2 μg) at 33°C (left panels) and 37°C (right panels) culture. β-actin was a normalization control.
(TIF)

S3 Fig Immunofluorescence analysis of type I IFN against HPeV1 infection. **(A)** Upper panels, immunofluorescence assay of 2×10^5^ A549 cells and **(B)** T84 cells treated with IFNα-2a or IFNβ (500 and 1000 U/ml) for 24 h before HPeV1 (MOI = 1) infection for 6 h. Lower panels, immunofluorescence assay with anti-VP0 antibody at 6 h post-infection with type I IFN added after HPeV1 adsorption.
(TIF)

S1 Table PCR primers for HPeV1 KVP6 sequencing. (DOC)

S2 Table Sequence for qPCR primers. (DOC)

S3 Table Human parechovirus 1 strain KVP6 polyprotein gene, complete cds, (GenBank: KC 769584). (DOC)

S4 Table HPeV nucleotide full-length similarity. (DOC)

S5 Table HPeV polyprotein similarity. (DOC)
