# Supplementary figures and images for: Genome and Infection Characteristics of Human Parechovirus Type 1: The Interplay between Viral Infection and Type I Interferon Antiviral System
Source: PLoS One. 2015 Feb 3;10(2):e0116158. doi: 10.1371/journal.pone.0116158 (PMC4380134; doi:10.1371/journal.pone.0116158)

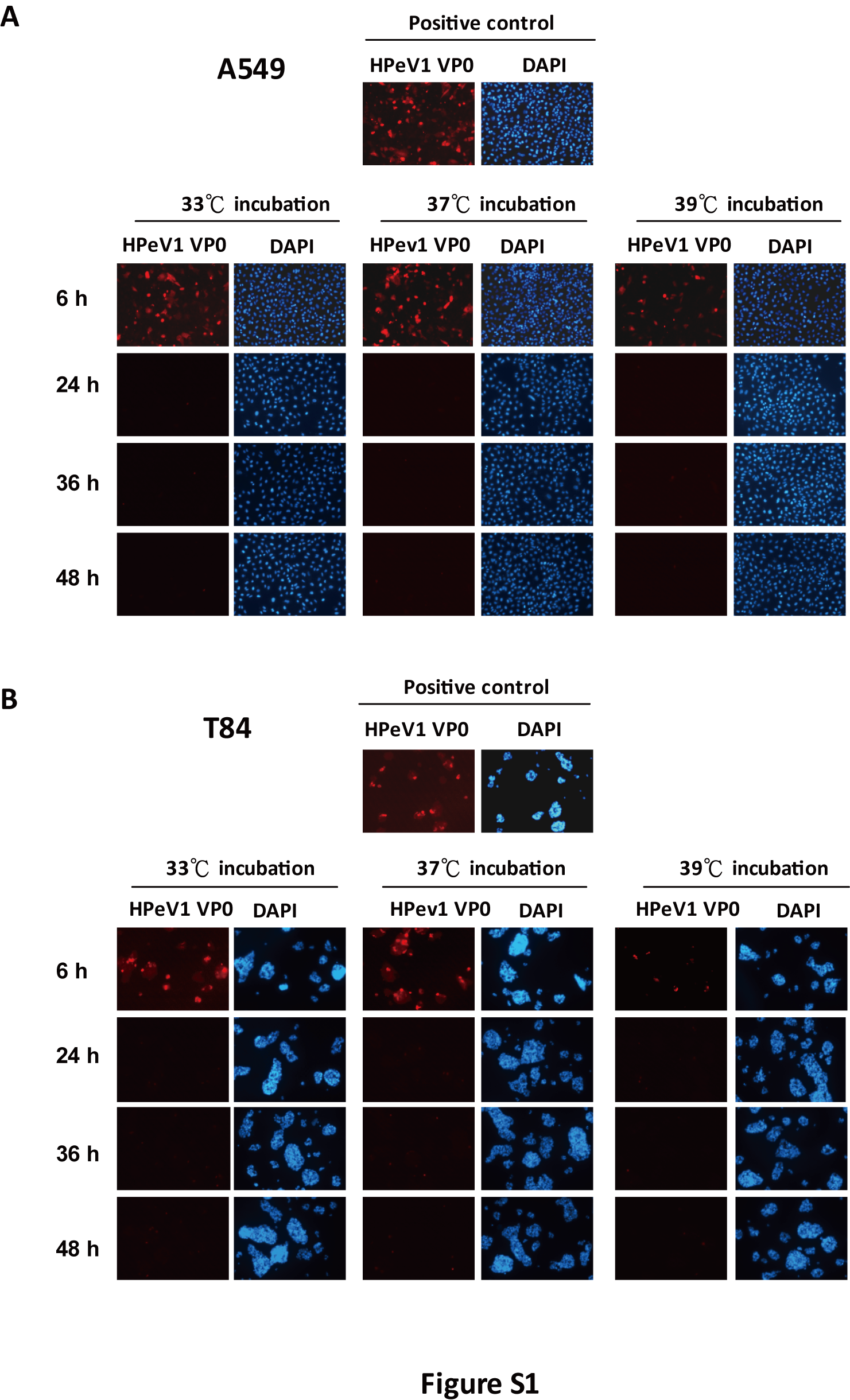

Supplement: S1 Fig — HPeV1 viral stocks were incubated at 33°C, 37°C and 39°C for 6, 24, 36 and 48 h before inoculation of A549 cells (A) and T84 cells (B). Immunofluorescence assay with anti-VP0 antibody at 6 h post-infection (hpi) of HPeV1 infection (multiplicity of infection [MOI] = 5). The positive controls (upper panels of A, B) are cells infected with HPeV1 without pre-incubation. (TIF) [file pone.0116158.s001.tif]

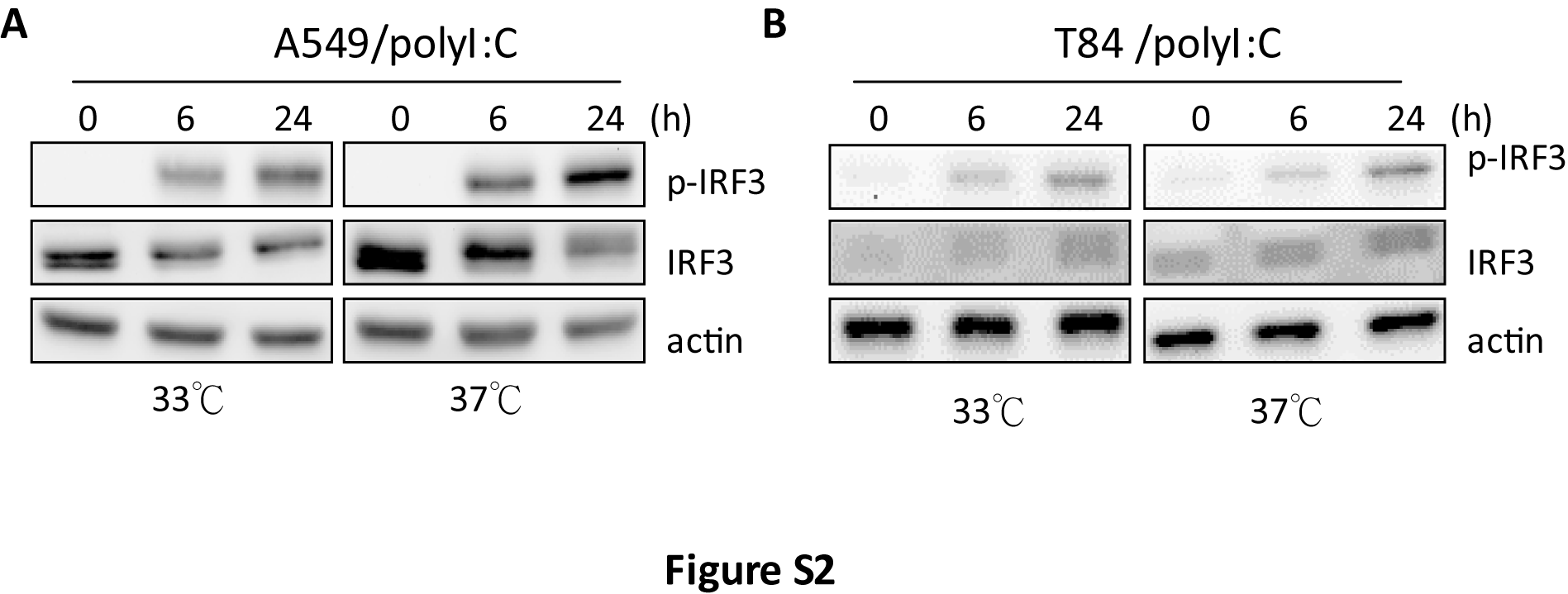

Supplement: S2 Fig — Immunoblotting analysis of phospho-and total IRF3 in 2×10^5^ A549 (A) or T84 cells (B) transfected with polyI:C (2 μg) at 33°C (left panels) and 37°C (right panels) culture. β-actin was a normalization control. (TIF) [file pone.0116158.s002.tif]

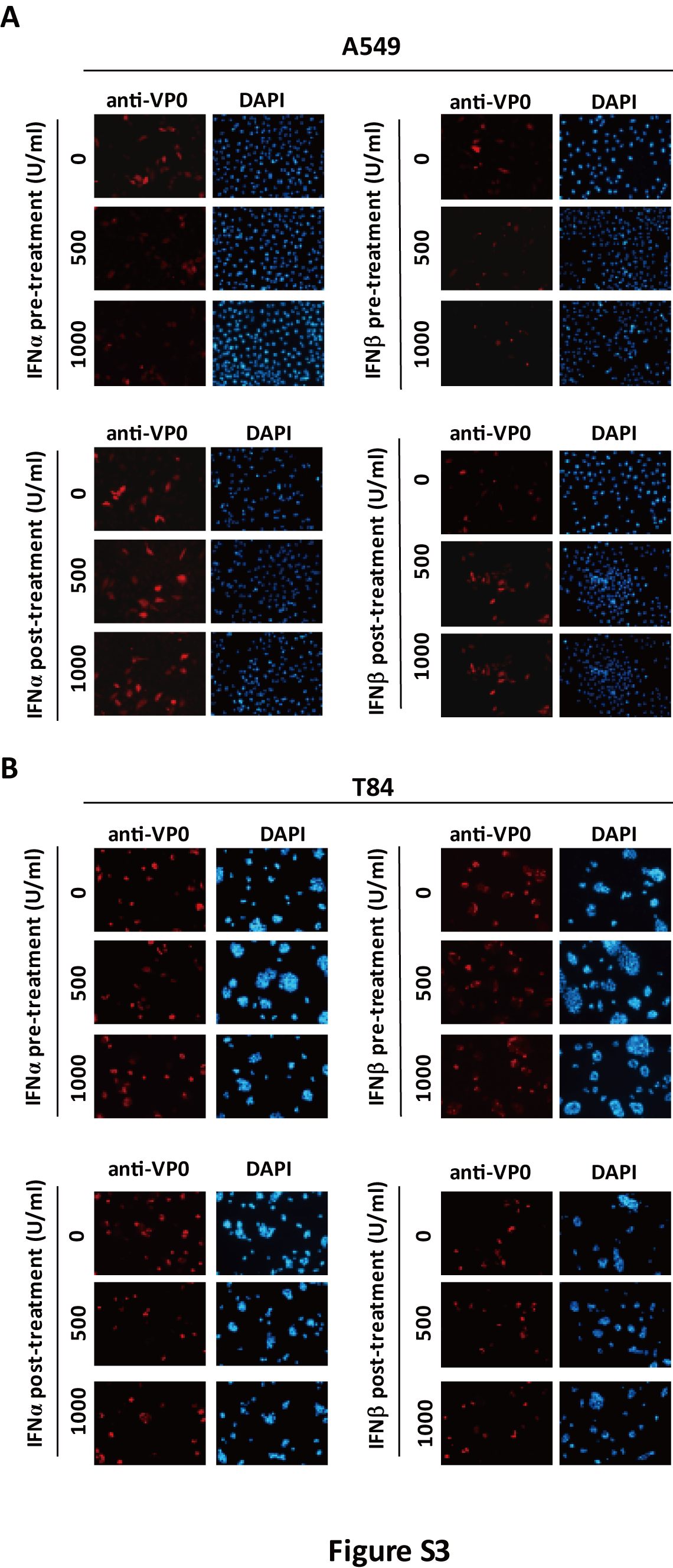

Supplement: S3 Fig — (A) Upper panels, immunofluorescence assay of 2×10^5^ A549 cells and (B) T84 cells treated with IFNα-2a or IFNβ (500 and 1000 U/ml) for 24 h before HPeV1 (MOI = 1) infection for 6 h. Lower panels, immunofluorescence assay with anti-VP0 antibody at 6 h post-infection with type I IFN added after HPeV1 adsorption. (TIF) [file pone.0116158.s003.tif]
