# Supplementary material for: Genome and Infection Characteristics of Human Parechovirus Type 1: The Interplay between Viral Infection and Type I Interferon Antiviral System
Source: PLoS One. 2015 Feb 3;10(2):e0116158. doi: 10.1371/journal.pone.0116158 (PMC4380134; doi:10.1371/journal.pone.0116158)
Supplement: S1 Table — (DOC) [file pone.0116158.s004.doc]

# Table S1. PCR primers for HPeV1 KVP6 sequencing

| Primer oligos | Sequences |
| --- | --- |
| 3’ RACE primer (RT) | 5'-GCTGTCAACGATACGCTACGTAACGGCATGACAGTG(T*14)-3' |
| Race 3' primer | 5'-GCTGTCAACGATACGCTACGTAACG-3' |
| HPeV1 nt 6342-endF | 5'-TGGTAAGACTAGGTGTATTGAAGCTTGTGA-3' |
| HPeV1 nt 5571-6401F | 5’-AAGTATGCTGATCTGGATGACTGAACAAGGT-3’ |
| HPeV1 nt 5571-6401R | 5’-AATAGTCCACTTCACAAGCTTCAATACACCTA-3’ |
| HPeV1 nt 4837-5641F | 5’-GATCTAGATGATGCTGTCTCGTACATCAAG-3’ |
| HPeV1 nt 4837-5641R | 5’-CGCCAGAATGATGAACTCTCTGAACTTCTTT-3’ |
| HPeV1 nt 3706-4932F | 5’-CAGTGTCTGATGGACGGTGACGTCAA-3’ |
| HPeV1 nt 3706-4932R | 5’-AATCAAAGTAGACATTTCAATGTTAAGATATTCGTCAATGTA-3’ |
| HPeV1 nt 2987-3788F | 5’-GTGCTTTGCGGGGTGACTTAGCAAAT-3’ |
| HPeV1 nt 2987-3788R | 5’-TTGATTTCTTCATCATCTGTGTTAGACATGGATTC-3’ |
| HPeV1 nt 1481-2449F | 5’-CTACACAAGTTGATGTAACTATACTA-3’ |
| HPeV1 nt 1481-2449R | 5’-CATCATCCTGAGCTGATGTTAAT-3’ |
| HPeV1 nt 1-858F | 5’-CGTCGGGCCTTATACCCCGACTT-3’ |
| HPeV1 nt 1-858R | 5’-ACTAGATGCGTCATCTGCGACTTTAGTTAATA-3’ |
