# Supplementary material for: Genome and Infection Characteristics of Human Parechovirus Type 1: The Interplay between Viral Infection and Type I Interferon Antiviral System
Source: PLoS One. 2015 Feb 3;10(2):e0116158. doi: 10.1371/journal.pone.0116158 (PMC4380134; doi:10.1371/journal.pone.0116158)
Supplement: S2 Table — (DOC) [file pone.0116158.s005.doc]

**Table S2. Sequence for qPCR primers**

| Primer oligos | Sequence |
| --- | --- |
| HPeV1 VP1(+) | Forward: 5’-CGACACATCACAAAACAGGGTTA-3’ |
|  | Reverse: 5’-ATCTGTTCTCCTGCTGGAATTGTAA-3’ |
| HPeV1 VP1(-) | Forward: 5’-ACCCTGTTTTGTGATGTGTCGTA-3’ |
|  | Reverse: 5’-TGTTTTGTTCTTGGCTGGAAGA-3’ |
| HPeV1 3C(+) | Forward: 5’-ATCATTCTGGCGGCATCAA-3’ |
|  | Reverse: 5’-TGTGTAGCTGATTGTTTTTGTGCTT-3’ |
| HPeV1 3C(-) | Forward: 5’-TGCCGCCAGAATGATGAAC-3’ |
|  | Reverse: 5’-CATGTTGATTTGGATGACTGAACA-3’ |
| HPeV1 3D(+) | Forward: 5’-CGGTTCCCGCAAAGATGA-3’ |
|  | Reverse: 5’-CTTCAAAAATTCCACCTCCAGTCT-3’ |
| HPeV1 3D | Forward: 5’-TGTAACTGCATAACAAGATCAGGTGAA-3’ |
|  | Reverse: 5’-GTGGGAGGCTGTGGAGGTT-3’ |
| GAPDH | Forward: 5’-TGCACCACCAACTGCTTAGC-3’ |
|  | Reverse: 5’-GGCATGGACTGTGGTCAT-3’ |
| IFNα1 | Forward: 5’-CCTCGCCCTTTGCTTTACTG-3’ |
|  | Reverse: 5’-GCCCAGAGAGCAGCTTGACT-3’ |
| IFNβ | Forward: 5’-AGGTAGTAGGCGACACTGTTCGT-3’ |
|  | Reverse: 5’-AGAAGCACAACAGGAGAGCAATT-3’ |
| Viperin | Forward: 5’-AACCAGCGTCAACTATCACTTCAC-3’ |
|  | Reverse: 5’-TTTTGGCTGTGTGGAAACAGAA-3’ |
| IRF7 | Forward: 5’-AAGAGCCTGGTCCTGGTGAAG-3’ |
|  | Reverse: 5’-TCGATGTCGTCATAGAGGCTGTT-3’ |
| PKR | Forward: 5’-TCTACGCTTTGGGGCTAAT-3’ |
|  | Reverse: 5’-AGATGATGCCATCCCGTAG-3’ |
| MxA | Forward: 5’-GCTACACACCGTGACGGATATGG-3’ |
|  | Reverse: 5’-CGAGCTGGATTGGAAAGCCC-3’ |
| MAVS | Forward: 5’-AAAACTGGCAGTATCTACCAAAAGC-3’ |
|  | Reverse: 5’-CAGTCAGCGTGTGGTTAGAAACAT-3’ |
