# Supplementary material for: Genome and Infection Characteristics of Human Parechovirus Type 1: The Interplay between Viral Infection and Type I Interferon Antiviral System
Source: PLoS One. 2015 Feb 3;10(2):e0116158. doi: 10.1371/journal.pone.0116158 (PMC4380134; doi:10.1371/journal.pone.0116158)
Supplement: S3 Table — (DOC) [file pone.0116158.s006.doc]

**Table S3. Human parechovirus 1 strain KVP6 polyprotein gene, complete cds**, (GenBank: KC 769584)*

| Viral gene | Nucleotide length (bp) | Putative activity [1] |
| --- | --- | --- |
| 5’-UTR | 681 | Replication function, Contains IRES for translation initiation |
| VP0 | 867 |  |
| VP3 | 756 | Like role in receptor binding and target for neutralizing antibodies |
| VP1 | 693 | Like role in receptor binding and target for neutralizing antibodies. RGD motif used for integrin binding |
| 2A | 429 |  |
| 2B | 387 |  |
| 2C | 987 | RNA binding protein, contained helicase domain,  Role in formation of replication complex |
| 3A | 351 |  |
| 3B | 78 | VPg protein templates RNA transcription after uridylylation |
| 3C | 582 | Trpsin-like protease cleaves polyprotein after translation |
| 3D | 1407 | RNA-dependent RNA polymerase Copies viral genome during replication |
| 3’-UTR | 111 | Presumed role in virus replication |

*HPeV1 strain KVP6 full length: 7,329 bp, ORF: 6,537 bp, polyprotein: 2,179 amino acids

Reference:

1. Harvala H, Simmonds P (2009) Human parechoviruses: biology, epidemiology and clinical significance. J Clin Virol 45: 1-9.
