# Supplementary material for: Genome and Infection Characteristics of Human Parechovirus Type 1: The Interplay between Viral Infection and Type I Interferon Antiviral System
Source: PLoS One. 2015 Feb 3;10(2):e0116158. doi: 10.1371/journal.pone.0116158 (PMC4380134; doi:10.1371/journal.pone.0116158)
Supplement: S4 Table — (DOC) [file pone.0116158.s007.doc]

**Table S4. HPeV nucleotide full-length similarity**

|  | HPeV1 KVP6 (KC769584) | HPeV1 Harris (L02971) | HPeV1 SH1 (FJ840477) | HPeV1 7555312 (FM178558) | HPeV2 Williamson (AJ005695) | HPeV3 Can82853-01 (AJ889918) | HPeV3 A308/99 (AB084913) | HPeV4 K251176-02 (DQ315670) | HPeV4 Fuk2005-123 (AB433629) | HPeV5 T92-15 (AM235749) | HPeV5 CT86-6760 (AF055846)[1] | HPeV6 NII561-2000 (AB252582) | HPeV6 2005-823 (EU077518) | HPeV7 PAK5045 (EU556224) | HPeV8 BR/217/2006 (EU716175) | Ljungan virus 87-012G (EF202833) |
| --- | --- | --- | --- | --- | --- | --- | --- | --- | --- | --- | --- | --- | --- | --- | --- | --- |
| HPeV1 KVP6 (KC769584) |  | 80 | 87 | 88 | 78 | 80 | 80 | 82 | 80 | 78 | 78 | 79 | 79 | 82 | 78 | 56 |
| HPeV1 Harris (L02971) |  |  | 80 | 80 | 78 | 78 | 78 | 78 | 77 | 79 | 78 | 80 | 80 | 77 | 79 | 56 |
| HPeV1 SH1 (FJ840477) |  |  |  | 85 | 78 | 80 | 80 | 81 | 79 | 78 | 80 | 80 | 80 | 80 | 78 | 56 |
| HPeV1 7555312 (FM178558) |  |  |  |  | 78 | 81 | 81 | 82 | 81 | 78 | 78 | 79 | 79 | 80 | 78 | 56 |
| HPeV2 Williamson (AJ005695) |  |  |  |  |  | 77 | 77 | 78 | 77 | 77 | 77 | 78 | 78 | 77 | 78 | 56 |
| HPeV3 Can82853-01 (AJ889918) |  |  |  |  |  |  | 96 | 82 | 80 | 77 | 78 | 78 | 78 | 82 | 77 | 55 |
| HPeV3 A308/99 (AB084913) |  |  |  |  |  |  |  | 82 | 80 | 77 | 78 | 78 | 78 | 81 | 77 | 56 |
| HPeV4 K251176-02 (DQ315670) |  |  |  |  |  |  |  |  | 85 | 79 | 80 | 78 | 78 | 82 | 78 | 56 |
| HPeV4 Fuk2005-123 (AB433629) |  |  |  |  |  |  |  |  |  | 78 | 78 | 78 | 79 | 80 | 78 | 55 |
| HPeV5 T92-15 (AM235749) |  |  |  |  |  |  |  |  |  |  | 85 | 78 | 78 | 77 | 78 | 56 |
| HPeV5 CT86-6760 (AF055846) |  |  |  |  |  |  |  |  |  |  |  | 78 | 78 | 78 | 77 | 56 |
| HPeV6 NII561-2000 (AB252582) |  |  |  |  |  |  |  |  |  |  |  |  | 97 | 77 | 79 | 56 |
| HPeV6 2005-823 (EU077518) |  |  |  |  |  |  |  |  |  |  |  |  |  | 77 | 78 | 56 |
| HPeV7 PAK5045 (EU556224) |  |  |  |  |  |  |  |  |  |  |  |  |  |  | 78 | 56 |
| HPeV8 BR/217/2006 (EU716175) |  |  |  |  |  |  |  |  |  |  |  |  |  |  |  | 56 |
| Ljungan virus 87-012G (EF202833) |  |  |  |  |  |  |  |  |  |  |  |  |  |  |  |  |

**Reference:**

1. Al-Sunaidi M, Williams CH, Hughes PJ, Schnurr DP, Stanway G (2007) Analysis of a new human parechovirus allows the definition of parechovirus types and the identification of RNA structural domains. J Virol 81: 1013-1021.
