# Supplementary material for: Genome and Infection Characteristics of Human Parechovirus Type 1: The Interplay between Viral Infection and Type I Interferon Antiviral System
Source: PLoS One. 2015 Feb 3;10(2):e0116158. doi: 10.1371/journal.pone.0116158 (PMC4380134; doi:10.1371/journal.pone.0116158)
Supplement: S5 Table — (DOC) [file pone.0116158.s008.doc]

**Table S5. HPeV polyprotein similarity**

|  | HPeV1 KVP6 (AGO028196) | HPeV1 Harris (AAA72291) | HPeV1 SH1 (ACV73067) | HPeV1 7555312 (CAQ76820) | HPeV2 Williamson (CAA06679) | HPeV3 Can82853-01 (CAI64373) | HPeV3 A308/99 (BAC23086) | HPeV4 K251176-02 (DQ315670) | HPeV4 Fuk2005-123 (BAG65816) | HPeV5 T92-15 (CAJ84483) | HPeV5 CT86-6760 (AAC79756) | HPeV6 NII561-2000 (BAF63403) | HPeV6 2005-823 (ABX79460) | HPeV7 PAK5045 (ACD80088) | HPeV8 BR/217/2006 (ACF60607) | Ljungan virus 87-012G (ABQ02688) |
| --- | --- | --- | --- | --- | --- | --- | --- | --- | --- | --- | --- | --- | --- | --- | --- | --- |
| HPeV1 KVP6 (AGO028196) |  | 93 | 96 | 97 | 88 | 88 | 88 | 91 | 90 | 88 | 88 | 89 | 89 | 88 | 88 | 47 |
| HPeV1 Harris (AAA72291) |  |  | 93 | 93 | 88 | 87 | 87 | 89 | 88 | 88 | 88 | 91 | 91 | 87 | 89 | 47 |
| HPeV1 SH1 (ACV73067) |  |  |  | 96 | 88 | 88 | 88 | 90 | 90 | 88 | 88 | 89 | 89 | 88 | 88 | 47 |
| HPeV1 7555312 (CAQ76820) |  |  |  |  | 88 | 88 | 88 | 91 | 90 | 88 | 88 | 89 | 89 | 88 | 88 | 47 |
| HPeV2 Williamson (CAA06679) |  |  |  |  |  | 85 | 85 | 87 | 87 | 85 | 86 | 87 | 88 | 85 | 87 | 47 |
| HPeV3 Can82853-01 (CAI64373) |  |  |  |  |  |  | 98 | 89 | 88 | 86 | 87 | 87 | 87 | 89 | 86 | 47 |
| HPeV3 A308/99 (BAC23086 |  |  |  |  |  |  |  | 89 | 88 | 86 | 86 | 86 | 86 | 89 | 86 | 47 |
| HPeV4 K251176-02 (DQ315670) |  |  |  |  |  |  |  |  | 97 | 90 | 90 | 88 | 88 | 88 | 88 | 47 |
| HPeV4 Fuk2005-123 (BAG65816) |  |  |  |  |  |  |  |  |  | 89 | 90 | 88 | 88 | 88 | 88 | 47 |
| HPeV5 T92-15 (CAJ84483) |  |  |  |  |  |  |  |  |  |  | 95 | 87 | 87 | 86 | 87 | 46 |
| HPeV5 CT86-6760 (AAC79756) |  |  |  |  |  |  |  |  |  |  |  | 87 | 87 | 86 | 87 | 46 |
| HPeV6 NII561-2000 (BAF63403) |  |  |  |  |  |  |  |  |  |  |  |  | 99 | 85 | 88 | 47 |
| HPeV6 2005-823 (ABX79460) |  |  |  |  |  |  |  |  |  |  |  |  |  | 86 | 89 | 47 |
| HPeV7 PAK5045 (ACD80088 |  |  |  |  |  |  |  |  |  |  |  |  |  |  | 86 | 47 |
| HPeV8 BR/217/2006 (ACF60607 |  |  |  |  |  |  |  |  |  |  |  |  |  |  |  | 47 |
| Ljungan virus 87-012G (ABQ02688) |  |  |  |  |  |  |  |  |  |  |  |  |  |  |  |  |
